# RISK FACTORS FOR ASTHMA: THE CONTRIBUTION OF THE BRAZILIAN NATIONAL RESEARCH ON HEALTH OF SCHOOLCHILDREN

**DOI:** 10.1590/1984-0462/;2019;37;4;00020

**Published:** 2019-10-10

**Authors:** Antonio Carlos Pastorino

**Affiliations:** a Faculty of Medicine, Universidade de São Paulo, São Paulo, SP, Brazil.

The National Survey on the Health of Schoolchildren (*Pesquisa Nacional de Saúde do Escolar* - PeNSE) is considered the largest study performed on schoolchildren in the country and is in its 3^rd^ edition (2009, 2012 and 2015). It is carried out by the Brazilian Institute of Geography and Statistics (*Instituto Brasileiro de Geografia e Estatística* - IBGE) in partnership with the Ministry of Health, and it aims to monitor the health conditions of students throughout the national territory. The survey consists of 120 structured questions on a smartphone, which are answered by the students themselves. It includes two questions about asthma that are similar to those developed in the International Study of Asthma and Allergies in Childhood (ISAAC) questionnaire, asking about: symptoms of wheezing and if you have been diagnosed with asthma in the last 12 months. In 2012, about 106,983 adolescents in the 8^th^ grade participated in the survey, and there was an estimated asthma prevalence of 23.2%. In 2015, this same survey included 102,072 students from public and private schools from all of the Brazilian states and the Federal District, and the estimated prevalence of asthma was 23.5% (95% CI 22.88 - 24.15), which shows that the effectiveness in controlling asthma among adolescents remains low.^1^ ^,^ ^2^

Asthma is one of the most prevalent chronic diseases in the world, especially among children and adolescents. Genetic determinants cannot be considered the only factors that cause it. Environmental aspects are important in the development and onset of asthma attacks and were assessed based on several questions included in this research. The authors of the article entitled “Factors associated with asthma in Brazilian adolescents: The National Survey on the Health of Schoolchildren (PeNSE)”, 2012,^3^ studied the demographic, socioeconomic, clinical, food and environmental characteristics that could be potentially associated with asthma in a multivariate analysis model and, as expected, a series of independent variables were positive as risk factors for a multifactorial disease such as asthma. The PeNSE study was not designed specifically for asthma, and many other issues regarding disease evolution or environmental and personal factors were not studied, especially with regard to being sensitized to the most common allergens in the air. Knowing the risk factors for asthma in the adolescent population is a first step for public health policies to be implemented at regional and national levels.
